# Misguided Transcriptional Elongation Causes Mixed Lineage Leukemia

**DOI:** 10.1371/journal.pbio.1000249

**Published:** 2009-11-24

**Authors:** Dorothee Mueller, María-Paz García-Cuéllar, Christian Bach, Sebastian Buhl, Emanuel Maethner, Robert K. Slany

**Affiliations:** Department of Genetics, University Erlangen, Erlangen, Germany; Loyola University, United States of America

## Abstract

Fusion proteins composed of the histone methyltransferase mixed-lineage leukemia (MLL) and a variety of unrelated fusion partners are highly leukemogenic. Despite their prevalence, particularly in pediatric acute leukemia, many molecular details of their transforming mechanism are unknown. Here, we provide mechanistic insight into the function of MLL fusions, demonstrating that they capture a transcriptional elongation complex that has been previously found associated with the eleven-nineteen leukemia protein (ENL). We show that this complex consists of a tight core stabilized by recursive protein–protein interactions. This central part integrates histone H3 lysine 79 methylation, RNA Polymerase II (RNA Pol II) phosphorylation, and MLL fusion partners to stimulate transcriptional elongation as evidenced by RNA tethering assays. Coimmunoprecipitations indicated that MLL fusions are incorporated into this complex, causing a constitutive recruitment of elongation activity to MLL target loci. Chromatin immunoprecipitations (ChIP) of the homeobox gene A cluster confirmed a close relationship between binding of MLL fusions and transcript levels. A time-resolved ChIP utilizing a conditional MLL fusion singled out H3K79 methylation as the primary parameter correlated with target expression. The presence of MLL fusion proteins also kept RNA Pol II in an actively elongating state and prevented accumulation of inhibitory histone methylation on target chromatin. *Hox* loci remained open and productive in the presence of MLL fusion activity even under conditions of forced differentiation. Finally, MLL-transformed cells were particularly sensitive to pharmacological inhibition of RNA Pol II phosphorylation, pointing to a potential treatment for MLL. In summary, we show aberrant transcriptional elongation as a novel mechanism for oncogenic transformation.

## Abstract 2

Investigation of the activity of a family of fusion proteins that cause aggressive leukemia suggests transcriptional elongation as a new mechanism for oncogenic transformation.

## Author Summary

The expression level of a gene needs to be precisely adjusted to ensure proper function. Adjustments can be imposed at different stages during the overall process of gene expression, including transcription initiation, transcript elongation, and transcript processing. If control of one of these mechanisms fails, aberrant gene expression can occur, which may have severe consequences such as cellular transformation and the development of cancer. Here, we show that a class of aberrant fusion proteins that are causal in mixed-lineage leukemia (MLL) hijacks a transcriptional elongation complex. We analyze the architecture of this transcriptional elongation complex and demonstrate that the complex is targeted by MLL fusion proteins to genes that should normally be silenced to allow maturation of hematopoietic cells. We show that this mistargeting causes constitutive expression of the respective genes, which likely leads to inhibition of blood cell differentiation at a precursor cell stage in which the cells are highly proliferative. Such abnormal precursor cells have been shown previously to be resistant to normal differentiation signals and to form the leukemia-initiating population. We further show here that cells carrying MLL fusion proteins are more sensitive to chemical inhibition of transcriptional elongation than leukemic cells of different etiology. Our results propose transcriptional elongation as a new oncogenic mechanism and point to a potential specific therapy for this hard-to-cure leukemia.

## Introduction

Mixed-lineage leukemia (MLL) is a particularly aggressive subtype of acute leukemia with a very dismal prognosis. This disease is caused by chromosomal aberrations, mostly translocations, affecting Chromosome 11 at band q23. This chromosomal locus contains the gene for the histone H3 lysine 4–specific methyltransferase *MLL* [1]–[4]. As a corollary of these genomic rearrangements the 5′ portion of *MLL* is fused in frame to a variety of different and mostly unrelated partner genes. The translation of the chimeric RNAs transcribed from the altered locus results in the production of fusion proteins. In these fusions, the original MLL methyltransferase activity is replaced by biological properties provided by the fusion partner. This creates novel oncoproteins that are potently transforming hematopoietic cells (for reviews, see [5]–[7]).

MLL fusions are aberrant transcription factors that induce ectopic expression of their respective target genes, and as a consequence, they block hematopoietic differentiation. Critical targets for MLL-induced transformation are the clustered *HOXA* homeobox genes and the gene for the HOX-dimerization partner MEIS1 [8]. Accordingly, a relative overexpression of *HOXA* and *MEIS1* transcripts is the characteristic hallmark of the MLL-specific gene expression profile [9],[10]. Despite this predominance of *HOX* expression, however, it has been shown by genome-wide chromatin precipitations that MLL fusion proteins occupy several thousand binding sites [11]–[13]. As it has been noted some time ago, transcriptional activation by MLL fusions is accompanied by a conspicuous and dramatic increase of histone H3 lysine 79 dimethylation across the *HOXA* locus [14], and this phenomenon has been confirmed also for many of the other MLL fusion target loci [12]. The only known histone methyltransferase that is capable of introducing the H3K79 mark is DOT1L, a protein conserved from yeast to man [15],[16]. Indeed, it could be shown for the MLL fusion partner AF10 that a direct interaction with DOT1L was instrumental for the oncogenic function of the respective fusion protein [17].

First hints for a shared function of several MLL fusion partners came from studies performed by Bitoun et al. [18]. These authors conducted overexpression studies and published data to support a model of multiple MLL fusion partners being involved in a transcriptional elongation complex that includes the MLL partner proteins AF4, AF9, ENL, and AF10, as well as DOT1L, and positive transcription elongation factor b (pTEFb). A direct interaction between proteins of the AF4 and AF9/ENL families had been noted before by Erfurth et al. [19], as well as our own group [20]. A somewhat contradictory interaction of AF9 and DOT1L has also been described to be necessary for aldosterone-induced gene silencing [21]. To elucidate the function of normal ENL, we recently purified wild-type ENL from mammalian nuclei [22]. It could be shown that endogenous ENL was also present in a large macromolecular protein complex similar to the one described by Bitoun et al [18]. Although the complex was initially termed ENL associated proteins (EAP), we now propose to redefine EAP as “elongation assisting proteins” to better reflect the function of this protein complex. In addition to DOT1L, EAP contained pTEFb, a cyclin-dependent kinase 9/cyclin T dimer that phosphorylates the C-terminal repeat domain (CTD) of RNA Polymerase II (RNA Pol II) [23]. CTD phosphorylation is necessary to ensure productive transcriptional elongation. Next to AF4, the AF4 homologs AF5 and LAF4 were also present in EAP, confirming the results of Estable et al. [24], who had copurified AF5 with pTEFb. AF4 itself is the most frequently encountered MLL fusion partner, and in a recent survey, approximately 50% of all MLL cases in infants and adults carried a *MLL-AF4* translocation [25]. EAP was ubiquitously expressed, and interference with EAP assembly affected transcriptional elongation of many genes. However, it was not clear whether EAP activity was important for the respective MLL fusion proteins. In the fusion, a bulky 180-kDa MLL moiety is added to an ENL protein of approximately 70 kDa. This type of modification might substantially alter or even destroy the EAP complex.

Here, we present a comprehensive picture of MLL fusion biology, demonstrating that EAP has a very stable core that is capable of also accommodating MLL fusion proteins. The constitutive recruitment of EAP to MLL target loci is responsible for persistent target transcription through stimulation of transcriptional elongation. This mechanism resists differentiation stimuli and therefore causes a maturation arrest. Finally, MLL fusion transformed cells were sensitive to EAP inhibition, pointing to a potential lead for pharmaceutical intervention.

## Results

### MLL Fusion Partners Are Constituents of an EAP Core Complex That Is Stabilized by Recursive Protein–Protein Interactions

**Figure 1:**
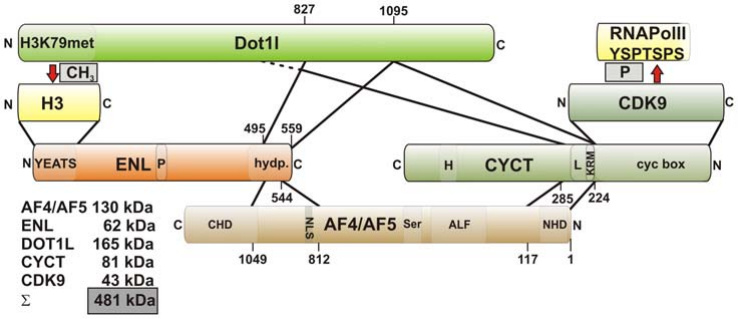
Core structure of the EAP complex. Schematic representation of the EAP core and stabilizing protein interactions. The composition of the EAP core, protein interaction motifs, and the enzymatic reactions catalyzed by EAP are depicted. Please note the individual protein orientation with N- and C-termini labeled appropriately. For clarity, only one RNA Pol II repeat sequence (YSPTSPS) is shown. Solid lines mark protein–protein interaction domains. The exact N-terminal extent of the CyclinT2 binding site in Dot1l could not be delineated, and therefore, this boundary is represented by a dashed line. The interaction of histone H3 with the YEATS domain of ENL has been described previously [20]. The calculated molecular weights from the respective reference sequences are given for each protein. Numbers denote the respective amino acid residues of the individual interaction domains. Abbreviations are used as follows: AF4, ALL1 fused gene on Chromosome 4 (also known as MLLT1 or AFF1); ALF, AF4-LAF4-FMR2 homology region; CDK9, cyclin dependent kinase 9; CHD, C-terminal homology domain; cyc box, cyclin box; CYCT, Cyclin T2; H, histidine rich; Dot1l, disruptor of telomeric silencing like; ENL, eleven nineteen leukemia; H3, histone H3; H3K79met, Histone H3 Lysine 79 methyltransferase domain; hydp, hydrophobic region; L, leucine rich; KRM, lysine-arginine-rich motif; NHD, N-terminal homology domain; NLS, nuclear localization signal; P, proline rich region; Ser, serine rich domain; YEATS, Yaf9, ENL, AF9, Taf14, and Sas5 conserved domain.

In previous studies, the total molecular weight of all proteins coprecipitating with ENL amounted to more than 1 MDa, whereas the bulk of ENL eluted on sizing columns with an apparent molecular weight of approximately 400 kDa to 500 kDa [22]. To explain this discrepancy and to further elucidate the molecular architecture of the EAP assembly, we performed two-hybrid assays to test for mutual protein–protein interactions. A large deletion series of existing [20],[22],[26],[27] and newly constructed two-hybrid bait clones for ENL, AF4, CYCT2A—the cyclin component of pTEFb—and Dot1l was probed for interaction with full-length versions of the same proteins. As reported previously [22], only the mouse homolog of DOT1L was available in cDNA repositories, and therefore, mouse Dot1l was used throughout this study. A total of 78 potential interaction pairs were interrogated (Figure S1). These experiments showed that EAP contained a tight core stabilized by a recursive set of direct protein–protein interactions (Figure 1). Each protein tested was able to interact with two other proteins, thus linking ENL, AF4, Dot1l, and CYCT2/CDK9 (pTEFb) in a tight “circular” network. In this way, histone H3 methylation catalyzed by Dot1l can be coordinated with RNA Pol II phosphorylation introduced by pTEFb. The total calculated molecular weight of the EAP core components was 481 kDa, and this number was very close to the previously determined [22] value for the EAP complex eluting from gel filtration. Of note, ENL, Dot1l, and CYCT2 utilized a single domain to interact with both of their binding partners, whereas the two binding interfaces were separated in AF4. The AF4 N-terminal homology domain provided contact to CYCT2, whereas sequences further C-terminal formed the interface with ENL. It is important to note that the respective interaction domains are highly conserved between the homologous MLL fusion partners ENL and AF9 and between AF4 and the related AF5, LAF4, and FMR2 proteins as well. In two-hybrid assays, AF4 sequences could be replaced with the corresponding AF5 regions, yielding identical results (unpublished data). In the cellular environment, EAP, therefore, is likely to exist in different configurations, explaining the large number of proteins that have been identified in ENL precipitates.

### MLL Fusion Partners Promote Transcriptional Elongation through Interaction with EAP Core Components

**Figure 2:**
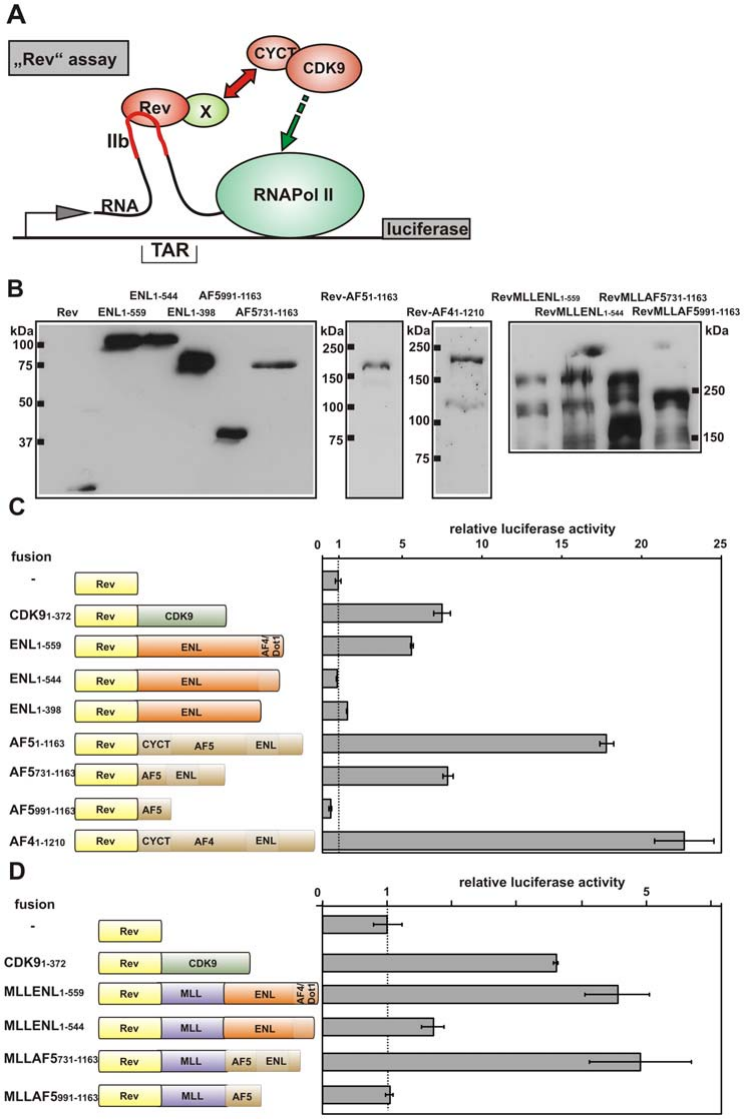
Elongation stimulation by EAP. (A) Mechanism of RNA tethering assay. A luciferase reporter driven by a modified HIV LTR promoter is used to detect elongation stimulation. The Rev RNA recognition site (IIb stem-loop) is grafted onto the TAR Tat-recognition loop. Proteins tethered to RNA via Rev will release the stalled RNA Pol II and create luciferase activity only if they can recruit active pTEFb (dimer of cyclin T1 or T2 and CDK9). (B) Expression of Rev fusion proteins. ENL, AF4, AF5, and derivatives thereof were fused to Rev and expressed in 293T cells. Cell lysates were probed with an antibody specific for Rev. For detection of Rev-MLL-fusion proteins, an anti-MLL antibody was employed. (C) Results of RNA tethering assays with MLL fusion partners. Rev or Rev fusions as indicated were expressed together with the TAR-IIb reporter and luciferase activity was determined. Boxes inside the graphical representation correspond to the protein–protein interaction domains from Figure 1. Values represent average and standard deviation of triplicate experiments and are expressed relative to background with Rev alone. (D) Elongation activity of MLL fusion proteins. Chimeras of Rev with MLL fusion proteins as depicted were tested in RNA tethering assays as described for (C).

Next, we wanted to know whether recruitment of MLL fusion partners to specific genes would promote transcriptional elongation. For this purpose, we used an RNA tethering assay to detect elongation activity. This test places a luciferase reporter gene downstream of a modified HIV-1 LTR promoter that grafts the stem loop IIb from the HIV-1 Rev response element (RRE) onto the TAR (transactivation response RNA) double-stranded RNA (Figure 2A). RNA Pol II stalls after the TAR element. LTR regulation is achieved by binding of the transactivator TAT that regulates promoter output via recruitment of pTEFb to stimulate elongation [28]. The hybrid IIb/TAR loop allows tethering of any protein of interest to the LTR RNA by fusing it to the RNA binding protein Rev. Luciferase levels, therefore, will reflect the ability to recruit pTEFb elongation activity [29].

Rev fusions with ENL, AF5, AF4, or deletion derivatives of these proteins were transiently expressed in 293T cells in the presence of the TAR/IIb luciferase reporter. Correct expression was verified by an anti-Rev immunoblot (Figure 2B). Rev alone and a Rev-CDK9 chimera served as negative and positive controls, respectively. Because pTEFb is ubiquitously expressed, 293T cells are a suitable environment for this assay. Attaching ENL to Rev induced an approximately 6-fold increase in luciferase levels comparable to the effect of a Rev-CDK9 fusion (Figure 2C). Because ENL does not directly interact with pTEFb (compare to Figure 1), this contact must have been made through endogenous AF4, Dot1l, or both proteins, respectively. Consequently, small deletions in the C-terminal AF4/Dot1l interaction domain eliminated Rev-ENL activity. As expected, Rev-AF5 and Rev-AF4 induced overall higher luciferase outputs because these molecules should be able to recruit pTEFb directly through CYCT binding and, in addition, indirectly via ENL. Deletion of the CYCT binding domain in AF5 should allow EAP interaction only through ENL. Indeed, a corresponding Rev-AF5 mutant had reduced luciferase activities comparable to the values achieved with Rev-ENL alone. No effect on elongation could be recorded with AF5 lacking both CYCT and ENL interaction regions. These results provided strong evidence that recruitment of MLL fusion partners induced elongation activity.

Next, we tested whether the elongation activity of ENL and AF5 persisted after fusion with MLL. MLL-ENL and MLL-AF5, as well as two mutants with a deletion in the respective EAP interaction domains, were joined to Rev and tested in the RNA tethering assay (Figure 2D). In these experiments, Rev-MLL-ENL and Rev-MLL-AF5 could activate the luciferase reporter to a similar degree as Rev-CDK9. In contrast, luciferase levels were close to background for the Rev-MLL fusions that had lost the capability to recruit EAP. This indicated a functional association also of MLL fusion proteins with EAP.

In the past, it has been noted by So and Cleary [30] and by our group [31] that a heterologous fusion of MLL with the strong transactivator VP16 had transforming ability. In contrast, chimeras of MLL and the acidic transactivation domain AD42 (derived from a mammalian two-hybrid system) had no oncogenic activity despite the fact that AD42 was a more powerful transactivator than ENL. Later, it was shown that VP16 recruits pTEFb [32], whereas no such activity is known for AD42. To further strengthen the correlation between elongation and functional MLL fusion proteins, we determined the overall transactivation capability of ENL, AD42, and VP16 in a conventional GAL4-based reporter assay and compared it to the elongation activity as Rev fusion (Figure S2). GAL4-ENL was 30-fold weaker than GAL4-VP16 and 5-fold less active than GAL4-AD42 in SV40 core promoter-based reporter assays. In stark contrast, Rev-ENL induced almost the same elongation activity as Rev-VP16 on the TAR-reporter, whereas Rev-AD42 showed almost no elongation stimulation in this test.

### MLL Fusion Proteins Are Incorporated into the EAP Core Structure

**Figure 3:**
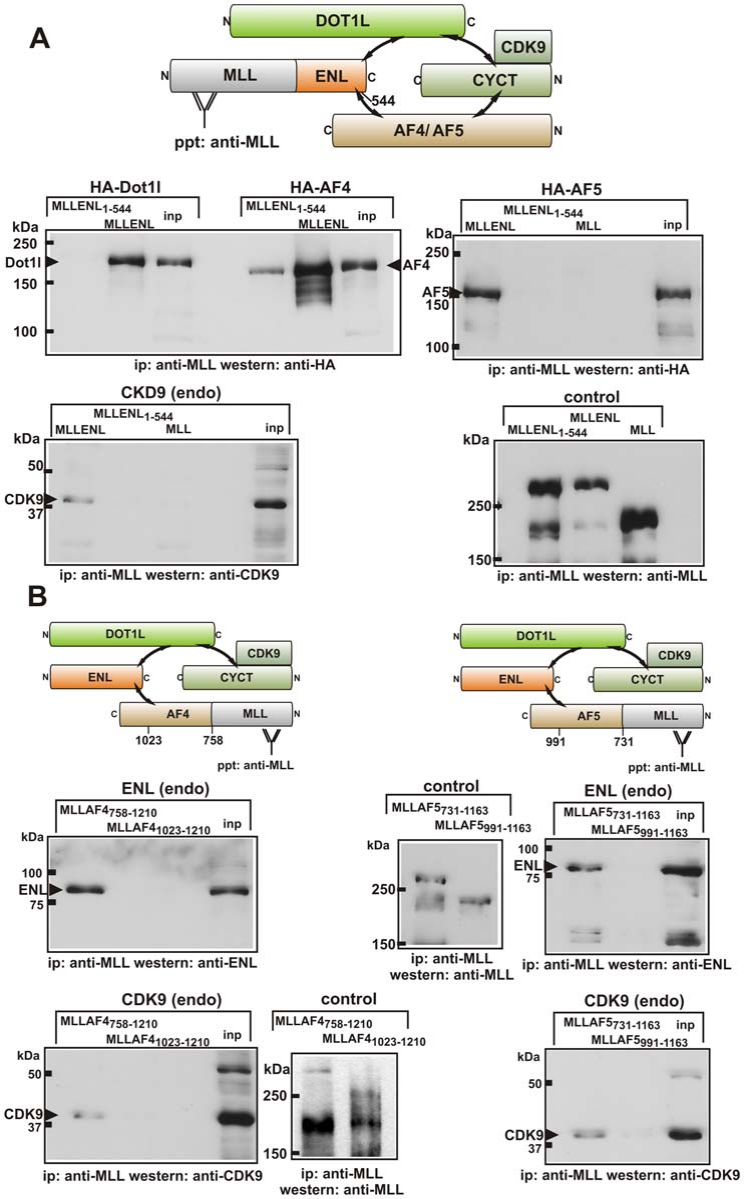
Incorporation of MLL fusion proteins into EAP. (A) Coimmunoprecipitation of MLL-ENL with EAP components. MLL-ENL (MLL-ENL), a MLL-ENL mutant lacking the last 15 amino acids of ENL (MLL-ENL_1–544_), or MLL without fusion partner (MLL) were expressed either alone or together with HA-tagged proteins AF4, AF5, or Dot1l. A schematic overview of the EAP core structure in the presence of MLL-ENL including the expected protein–protein interactions (double-headed arrows) is depicted in the upper-left panel. The presence of HA-tagged proteins and endogenous CDK9 in anti-MLL precipitates was probed alongside with a sample of the input (inp, 5%) by immunoblots as indicated. As a control, the successful precipitation of the MLL-ENL derivatives was confirmed by an anti-MLL blot. (B) Interaction of MLL-AF4 and MLL-AF5 with endogenous (endo) ENL and CDK9. MLL-AF4 (MLLAF4_758–1210_) and MLL-AF5 (MLLAF5_731–1163_) proteins analogous to patient-derived fusions were expressed in 293T cells. Note that the N-terminal CYCT interaction domain is missing in leukemogenic MLL-AF4/5 fusions as depicted in the upper-left and -right panels. MLL-AF4/5 derivatives deleting the ENL binding domain (MLLAF4_1023–1210_, MLLAF5_991–1163_) served as controls. The coprecipitation of endogenous ENL and CDK9 was detected by immunoblot as indicated next to a sample of 5% input (inp). The presence of MLL-AF4/AF5 in the lysates was controlled as above.

MLL fusion proteins add a large 180-kDa MLL moiety to the respective fusion partner. Therefore, it was not clear whether these huge proteins could be accommodated within the EAP core. To answer this question, a series of immunoprecipitations were performed. Because sensitive antibodies that detect their cognate antigen at endogenous levels were only available for ENL and CDK9 (pTEFb), HA-tagged versions of Dot1l, AF4, and AF5 were utilized for these experiments. MLL-ENL was transfected either alone or together with HA-Dot1l, HA-AF4, or HA-AF5 into HEK293T cells. MLL without any fusion partner and a MLL-ENL variant lacking the last 15 amino acids of ENL (MLLENL_1–544_) served as controls. As shown before, this deletion prohibited interaction of ENL with Dot1l in two-hybrid tests and abolished ENL-mediated elongation activity in the RNA tethering experiments. Western blots proved all MLL fusion derivatives to be correctly expressed (unpublished data). Precipitations were done employing an anti-MLL antibody [33] recognizing the MLL N-terminus retained in the fusion proteins (Figure 3A). MLL-ENL coprecipitated with HA-AF4, HA-AF5, HA-Dot1l, and notably also with endogenous CDK9. Because there is no direct interaction of ENL with CDK9 or CYCT (see Figure 1), MLL-ENL most likely had to be associated also with endogenous AF4/DOT1L to bring down CDK9. In line with the two-hybrid and RNA tethering results, the MLL-ENL_1–544_ mutation eliminated coprecipitation with HA-Dot1l and CDK9, but still allowed some residual interaction with AF4. Interestingly, this was not true for AF5 as this protein could not be detected in MLL-ENL_1–544_ precipitates. No protein precipitated with the N-terminus of MLL; therefore, all interactions must have been mediated by the respective fusion partner. As a control, all immunoprecipitates were also checked for the presence of the respective MLL fusion by an MLL-specific Western blot.

In a second series of immunoprecipitations, we concentrated on the interaction of MLL-AF4 and MLL-AF5 with endogenous proteins (Figure 3B). MLL-AF4/5 fusions that occur “naturally” in leukemia join MLL to a C-terminal portion of AF4/5. Therefore, these proteins do not contain the N-terminal cyclin interaction domain of AF4/5, but they retain the ENL interaction motif. MLL-AF4 and MLL-AF5 fusions built analogous to the patient-derived proteins (MLLAF4_758–1210_, MLLAF5_731–1163_) were expressed in HEK293T cells. Shortened constructs deleting also the respective ENL interaction domains (MLLAF4_1023–1210_, MLLAF5_991–1163_) served as controls. MLLAF4_758–1210_ and MLLAF5_731–1163_ both efficiently coprecipitated with endogenous ENL and CDK9. This interaction was not mediated by the MLL portion of the fusion, because the control proteins lacking the ENL binding domain were not capable of bringing down ENL or CDK9. In summary, these results provided proof that despite their considerable size, MLL fusions could be accommodated within the EAP core complex without disturbing the stabilizing protein interaction network.

### MLL Fusions Associate with EAP in Leukemia Cells

**Figure 4:**
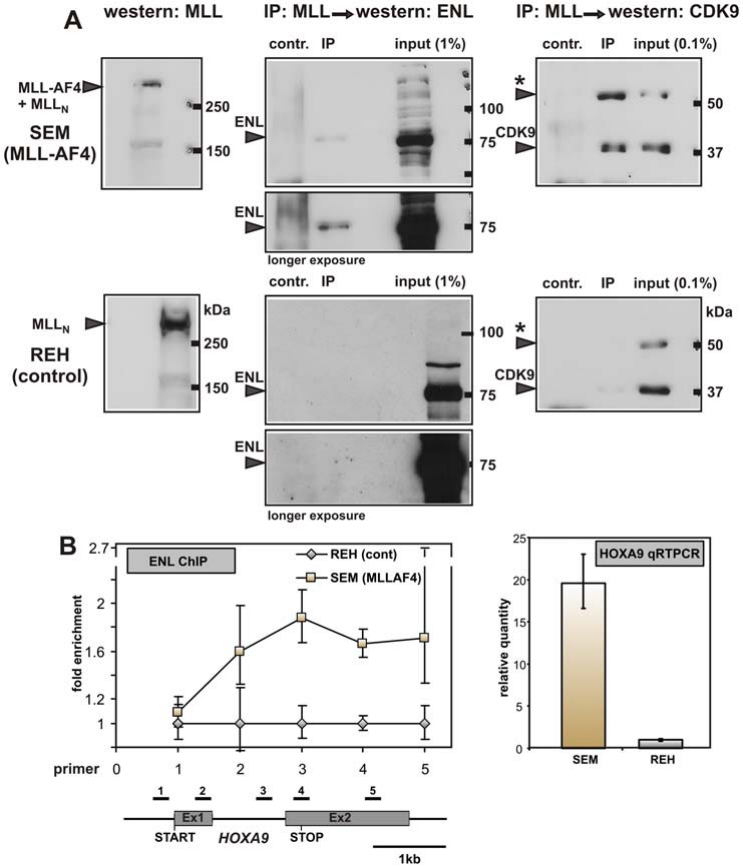
Association of MLL fusion proteins with EAP in leukemia cells. (A) Coimmunoprecipitation (IP) of MLL-AF4 with endogenous ENL and CDK9 in patient-derived SEM cells. The MLL-AF4 fusion protein was precipitated by anti-MLL antibodies from extracts of the t(4;11) B-lymphocytic leukemia line SEM (upper panels). To control for precipitation with wild-type MLL, a B-ALL line of different etiology (REH) was included (lower panels). Precipitates were analyzed alongside a sample of input and probed for ENL and CDK9. Mock precipitations without antibody served as additional controls (contr). Note that MLL-AF4 corresponds in size to the N-terminal part of MLL that is produced after posttranslational cleavage of wild-type MLL. Therefore, MLL-AF4 and MLL-N comigrate as single band. In SEM as well as in REH, a longer splice variant [58] of CDK9 (labeled by an asterisk [*]) also was prominently detectable. (B) Chromatin immunoprecipitation and *HOXA9* expression in SEM and REH cells. Left panel: ENL specific ChIP was performed across the human *HOXA9* locus in SEM and REH cells. Precipitation efficiencies relative to nonenriched input samples were determined for five locations across the human *HOXA9* region by quantitative PCR analysis of ENL bound chromatin in SEM (brown squares) and REH (grey diamonds). Values are plotted as relative enrichment with results from REH normalized to one unit. The experiment was performed three times with one typical example shown. Given are averages and standard deviations of triplicate PCR measurements. Right panel: *HOXA9* expression levels were determined by qRT-PCR from SEM and REH total RNA, normalized to actin, and plotted with REH as calibrator representing one unit.

To confirm the incorporation of MLL fusions into EAP also in authentic leukemia cells, the immunoprecipitation experiments were repeated with SEM cells, a B-ALL line transformed by MLL-AF4 [34]. Lymphoid REH cells without 11q23 translocation served as control (Figure 4A). Anti-MLL precipitates from SEM contained ENL and CDK9, corroborating the association of MLL fusion proteins with EAP. Because the MLL-AF4 protein from SEM cells does not encompass the CYCT interaction motif of AF4, the coprecipitation of CDK9 and MLL-AF4 strongly suggests an indirect bridging of these proteins by ENL and DOT1L. An association with the N-terminal MLL moiety or a nonspecific binding to the immunoprecipitation reagents seemed unlikely, as precipitates from REH cells done under identical conditions were devoid of ENL and CDK9.

The recruitment of EAP by MLL-AF4 should be accompanied by a higher concentration of EAP components on target chromatin. To test this prediction, the distribution of ENL across the human *HOXA9* locus, a known MLL target gene, was determined by ChIP in SEM and REH control cells (Figure 4B). Indeed, a significantly higher amount of ENL could be detected across the transcribed region of the *HOXA9* gene in SEM versus REH. This correlated well with an approximately 20-fold increased concentration of *HOXA9* RNA in SEM cells compared to REH controls.

### MLL Fusion Presence Determines Target Gene Expression

**Figure 5:**
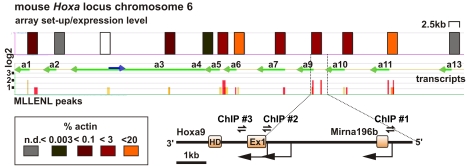
Correlation of MLL-ENL binding and *Hox* gene expression. Murine cell lines derived from primary hematopoietic cells by transformation with flag-MLL-ENL were subjected to a flag-specific ChIP reaction. Precipitates were amplified and hybridized (ChIP-chip) to a commercial promoter array that tiles 2 kb upstream and 0.5 kb downstream of known transcription start sites. In addition, the expression level of every *Hoxa* gene was determined by qRT-PCR with calibrated primers. Values were normalized to actin. The results are included as a heat map in the upper line (array set-up/expression level) of the figure. The heat map legend is shown below the figure. Each color-coded rectangle corresponds to 2.5 kb of genomic sequence represented on the promoter array. The second line shows known transcripts within the *Hoxa* locus. All *Hoxa* transcripts (green arrows drawn to scale) are transcribed in centromeric direction. The blue arrow denotes an annotated cDNA (5730596B20RIK-201 in Ensembl) transcribed oppositely to the *Hox* genes. The complete locus as shown encompasses 95 kb of genomic DNA. The third line represents a diagram of flag-MLL-ENL binding. The bar height indicates the log_2_ ratio of relative enrichment compared to a control precipitation. The bar color indicates a false discovery probability of <0.05 (red), <0.1 (orange), and <0.2 (yellow). The graph is a composite image compiled from two independent experiments. The genomic environment between *Hoxa9* and the gene for microRNA196b is shown enlarged. Arrows represent known transcript start sites, and double arrows indicate the location of the primer pairs used for the kinetic analysis described in Figure 6. n.d. = not detected.

To study the consequences of MLL fusion-mediated EAP recruitment for target chromatin, we first determined the binding sites of an MLL-ENL fusion across the *HoxA* locus by chromatin immunoprecipitation (ChIP) and hybridization to genomic arrays (ChIP-chip). For this purpose, MLL-ENL-transformed cell lines were generated from primary murine hematopoietic cells by transduction with a flag-tagged version of MLL-ENL. These fMLL-ENL cells were used as starting material for a flag-specific ChIP to avoid cross detection of endogenous wild-type Mll or Enl. Precipitates were amplified by ligation-mediated PCR and hybridized to commercial promoter arrays that tile 2.5 kb of genomic sequence upstream and downstream of the start of all known reading frames. In addition, the expression level of every single *Hoxa* gene was determined by quantitative reverse-transcriptase PCR (qRT-PCR) (Figure 5). With the exception of *Hoxa2* and *Hoxa13*, all *Hoxa* genes could be detected in fMLL-ENL cells with expression levels in the order *Hoxa6/11*>*Hoxa5/7/9/10*>*Hoxa1/3*≫*Hoxa4*. A close correlation was observed between fMLL-ENL bound upstream of the individual *Hoxa* genes and the presence of the respective transcript, suggesting an involvement of the fusion protein in control of *Hoxa* transcription.

### MLL Fusion-Mediated EAP Recruitment Catalyzes Highly Dynamic Chromatin Modifications

**Figure 6:**
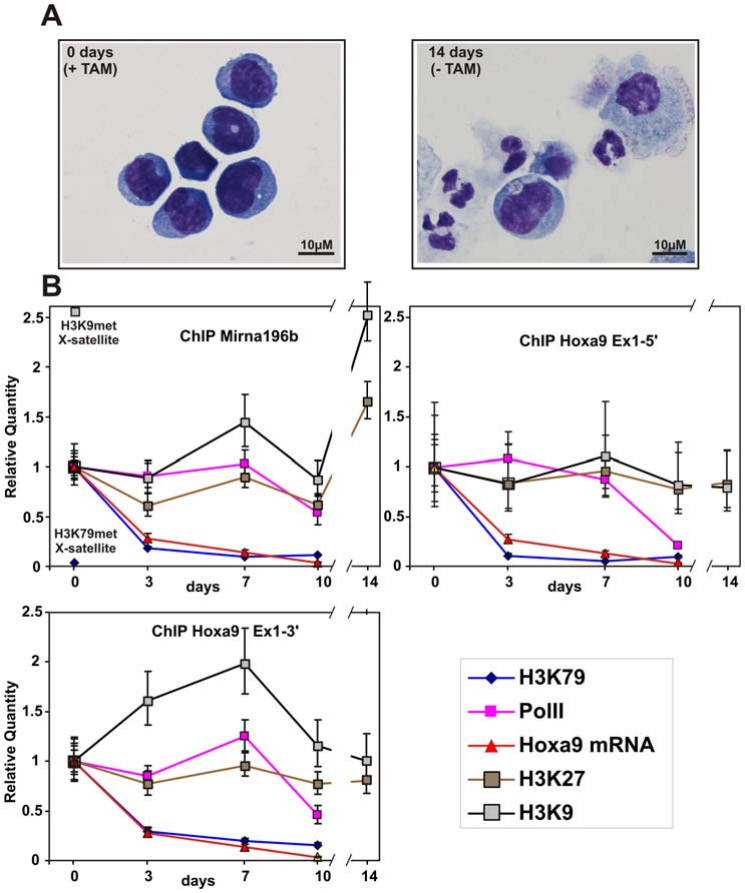
Dynamic modification of the *Hox* locus by MLL fusion-recruited EAP. (A) Morphology of cells transformed by a conditional derivative of MLL-ENL. Cytospin samples of cells transformed by tamoxifen-inducible MLL-ENL (MLL-ENL-ER as described in [8]) were prepared in the presence of tamoxifen (0 days) (+TAM) and 2 wk after withdrawal of inductor (−TAM). Slides were stained with May-Grünwald-Giemsa, and microphotographs were taken at room temperature with a Nikon Digital Sight DS-Fi1 electronic camera attached to a Zeiss Axioskop with a Zeiss Neofluar 63×/1.25 objective and processed with CorelDraw software (Corel) without any image enhancements. (B) ChIP experiments. MLL-ENL-ER cells as in (A) were used to record the histone H3 methylation status at lysines 9 (dimethylation, black line), lysine 27 (di- and trimethylation, brown line), and lysine 79 (dimethylation, blue line) as well as RNA Pol II presence (Ser2 phosphorylated polymerase, magenta line). ChIP samples were analyzed for two loci within the first exon of *Hoxa9* and upstream of the gene for microRNA196b. Samples were drawn at day 0 (active MLL-ENL) as well as 3, 7, 10 and 14 d after inactivation of MLL-ENL by inductor withdrawal. Precipitation efficiencies were normalized to an input sample, and values were plotted relative to the modification levels at day 0. For absolute comparison, H3K9 trimethylation and H3K79 dimethylation were also determined for a nontranscribed X-chromosomal satellite repeat sequence (indicated in upper-left panel). In addition, changes in *Hoxa9* mRNA after MLL-ENL-ER shutdown were quantified by qRT-PCR and plotted alongside the ChIP results (red line). The chart shows average values and standard deviations of a triplicate qPCR evaluation, and represents one typical of three independent experiments.

To get further insight into the molecular mechanism of gene regulation by MLL-ENL, we analyzed the genomic region upstream of *Hoxa9*, including the newly identified gene for microRNA196b [35], by a time-resolved ChIP. Primers were designed binding upstream of *Mirna196b* and at the 5′ as well as the 3′ ends of the first exon of *Hoxa9*. ChIP was done with a cell line transformed by a conditional version of MLL-ENL [8]. In these cells, MLL-ENL is fused to a mutated estrogen receptor ligand binding domain. As a consequence, the oncogene is only active in the presence of the inductor tamoxifen (TAM). Removal of TAM leads to a loss of MLL-ENL binding within 72 h, down-regulation of *Hox* gene expression, cellular differentiation, and growth arrest [8],[14]. Approximately 2 wk after withdrawal of TAM, the cultures consisted mainly of mature granulocytes and macrophages (Figure 6A). The kinetics of *Hoxa9* transcript levels, H3K79 dimethylation, RNA Pol II occupancy, and the presence of inhibitory H3K9/H3K27 methylation after MLL-ENL shut-down was determined by ChIP and qRT-PCR (Figure 6B). In the presence of MLL-ENL (time point 0 days), activating H3K79 dimethylation of *Mirna196b* was 50-fold higher and repressive H3K9 dimethylation was 2.6-fold lower compared to a heterochromatic, nontranscribed satellite locus on the X chromosome. Loss of MLL-ENL function was followed by a reduction of *Hoxa9* transcripts to approximately 20% within 3 d, and a further drop below detection threshold was observed at day 10. Most strikingly, the decrease in *Hoxa9* transcripts was exactly replicated by H3K79 dimethylation, but not by RNA Pol II occupancy. Whereas H3K79 dimethylation was removed within 3 d, RNA Pol II did not exit the locus till day 10 after TAM withdrawal. This observation strongly suggests that Pol II became unproductive in the absence of active MLL-ENL. Inhibitory H3K9 and H3K27 methylation could only be detected at the *Mirna196b* locus after prolonged differentiation for 14 d. The transcriptional landscape of the *Hoxa* locus is complex, and it is not known where the *Hoxa9* transcript is initiated and where it terminates. Nevertheless, we used the currently available information (*Hoxa9* Ensembl mRNA: ENSMUST00000048680) to design primer pairs at the utmost 5′ and 3′ ends of this putative core transcript. The ChIP kinetics was repeated with antibodies specific for the serine-2 and serine-5 phosphorylated isoforms of RNA Pol II. Furthermore, mRNA levels were quantified by RT-PCR as a function of time at the 5′ and 3′ ends of the transcript and in addition with primers spanning the *Hoxa9* intron (Figure S3).

Serine-2 phosphorylated (elongating) RNA Pol II decreased faster at the 3′ than at the 5′ end of the chromatin corresponding to the *Hoxa9* core transcript. There was no significant difference in the mRNA decay kinetics if measured at the 5′ or 3′ terminus. However, the amount of spliced RNA as detected by intron-spanning primers leveled off more rapidly. This would be consistent with the known function of serine-2 phosphorylated RNA Pol II as “landing pad” for RNA processing enzymes. Finally, serine-5 phosphorylated (initiating) RNA Pol II stayed relatively constant with a tendency to exit first at the 5′ and later at the 3′ end. Whereas all these results are consistent with a function of pTEFb in MLL-ENL-mediated *Hoxa9* activation, a more far-reaching interpretation will have to await the exact knowledge of all existing *Hoxa9* transcripts.

**Figure 7:**
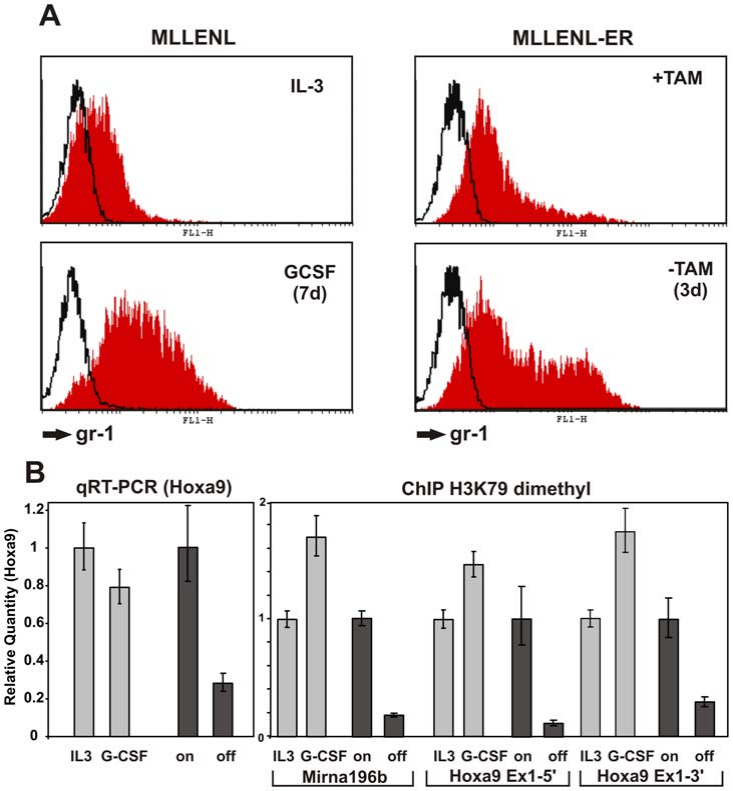
MLL-ENL-introduced chromatin modifications resist differentiation stimuli. (A) Left panel: cells transformed by constitutive MLL-ENL. FACS analysis of gr-1 differentiation marker in cells grown in IL-3 and in G-CSF for 7 d. Right panel: cells transformed by conditional MLL-ENL-ER. Gr-1 levels before and 3 d after inactivation of MLL-ENL-ER by removal of tamoxifen (TAM). (B) Left panel: qRT-PCR analysis of *Hoxa9* expression in cells transformed by constitutive MLL-ENL cultured in IL-3 or G-CSF for 7 d (light columns) and in cells transformed by conditional MLL-ENL-ER before and 3 d after tamoxifen removal (dark columns, labeled “on” and “off”). Right panel: comparison of H3K79 dimethylation status of the *Hoxa9* locus in cells treated as before. Values are averages and standard deviations of a triplicate, and the experiment was done twice with comparable results.

A second cell line model was used to confirm that MLL-ENL rather than cellular differentiation is responsible for the observed changes at the *Hoxa9* locus. It had been shown previously that treatment with G-CSF induces differentiation even in the presence of constitutively active MLL-ENL [36],[37]. MLL-ENL-transformed cells cultured in G-CSF will therefore allow separating the effect of MLL-ENL on the *Hoxa9* locus from the influence of cellular differentiation. For this purpose, H3K79 dimethylation and *Hoxa9* expression were determined in primary cells transduced by MLL-ENL subjected to G-CSF treatment. These data were compared to those measured in MLL-ENL-ER cells after MLL-ENL shutdown. Cells transformed by constitutive MLL-ENL stopped proliferation and induced gr-1 lineage marker expression after 7 d of G-CSF treatment to a level comparable to conditional MLL-ENL-ER cells 3 d after TAM withdrawal (Figure 7A and unpublished data). Despite these clear signs of differentiation, *Hoxa9* levels in G-CSF-cultured cells remained almost stable, and H3K79 dimethylation even increased slightly (Figure 7B), proving that MLL-ENL is directly responsible for these effects and that this molecule is able to override differentiation stimuli.

### MLL Fusion-Transformed Cells Are Sensitive to CDK9 Inhibition

**Figure 8:**
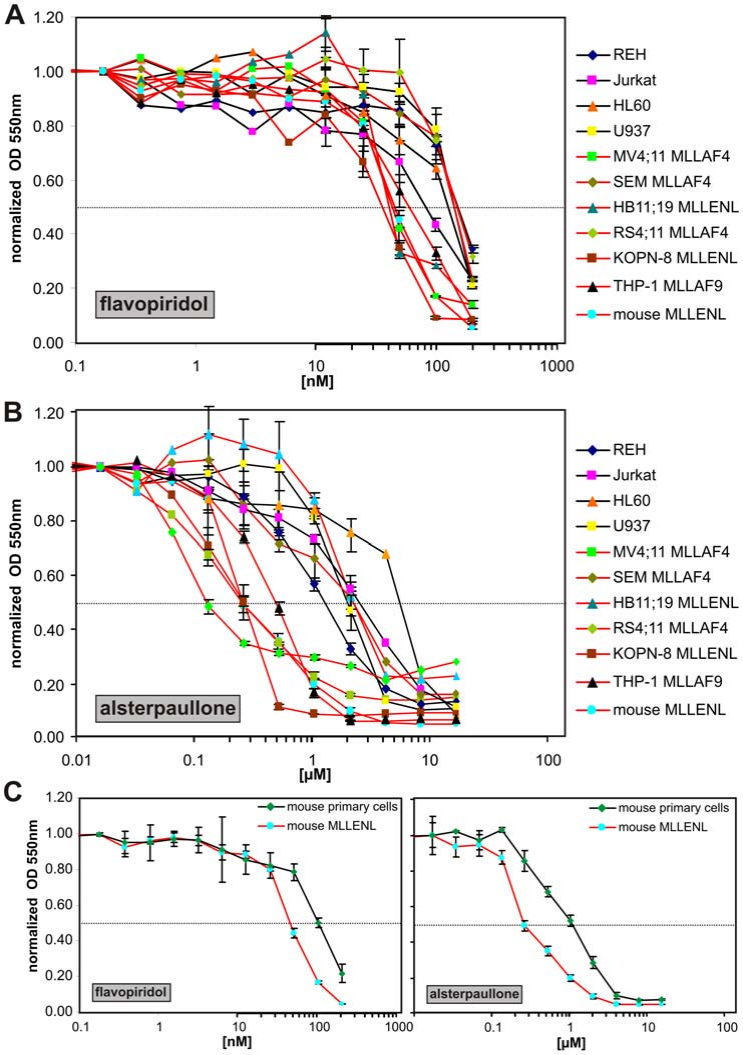
Sensitivity of MLL cells to CDK inhibition. (A) Effect of flavopiridol. Top panel: seven MLL cell lines (red lines) and four control leukemia lines of different etiology (black lines) were treated with increasing concentrations of flavopiridol as indicated. Proliferation was photometrically assessed in triplicate samples by MTT reduction. For better comparison, all optical density at 550 nm (OD_550_) readings were normalized to yield one unit in the absence of inhibitor. Mean values are plotted semilogarithmically against flavopiridol concentrations. To avoid clutter, standard deviations are indicated only for drug concentrations where MLL and control cell lines diverge in response to treatment. (B). Effect of alsterpaullone. Alsterpaullone was tested like described for (A). (C) Effect of flavopiridol and alsterpaullone on primary hematopoietic cells. Triplicate samples of mouse bone marrow cells enriched in early precursors by 5-fluorouracil treatment of the donors were cultured in liquid medium supplemented with IL-3, GM-CSF, IL-6, SCF, and the indicated amount of flavopiridol or alsterpaullone (black line). Proliferation was measured by MTT reduction. For comparison, precursor cells were retrovirally transduced with MLL-ENL, and the resulting lines were tested again for drug sensitivity under identical culture conditions (red line). Given are mean values and standard deviations of triplicates.

All results obtained so far indicated that MLL fusion proteins transform through recruitment of the EAP-associated enzymatic activities. Therefore, MLL cells might be sensitive to a pharmacologic inhibition of EAP. To test this prediction, the proliferation of six MLL cell lines and four controls of different etiology was recorded in the presence of increasing concentrations of flavopiridol and alsterpaullone, two substances with known CDK inhibitory activity [38],[39] (Figure 8). The study was restricted to CDK inhibition as currently, there is no H3K79 methyltransferase inhibitor available. A murine cell line experimentally transformed by MLL-ENL and the corresponding parental primary cells were also included in the assay because patient lines might have accumulated unknown additional mutations that render the cells more resistant to EAP inhibition. Plotting proliferation against inhibitor concentrations clearly separated the cells into a sensitive and a more resistant class with a cutoff value for the two groups at 50% inhibitory concentrations (IC_50_s) of approximately 80 nM for flavopiridol and 1 µM for alsterpaullone. Although two MLL lines fell within the more resistant group (RS4;11 and SEM for flavopiridol; HB11;19 and SEM for alsterpaullone), the majority of MLL fusion-transformed cells reacted significantly more sensitively than the controls. MLL-ENL-transformed primary cells had anIC_50_ of 50 nM for flavopiridol and 0.3 µM for alsterpaullone, whereas nontransduced primary bone marrow cells grown in liquid culture had significantly higher ID_50_ values of approximately 100 nM for flavopiridol and 1 µM for alsterpaullone (Figure 8C). This confirmed that MLL-transformed cells are particularly sensitive to these substances.

## Discussion

**Figure 9:**
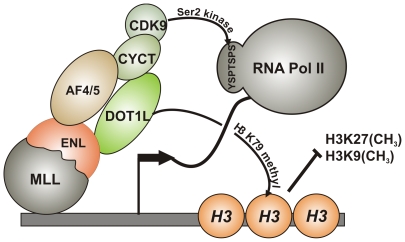
The molecular mechanism of transcriptional activation by MLL fusion proteins. MLL fusions recruit EAP core components and cause H3K79 methylation as well as Pol II CTD phosphorylation at serine 2. As a consequence, target genes are continuously expressed, inhibitory H3K27 and H3K9 methylation does not take place, and hematopoietic precursors are blocked for differentiation. The amino acid sequence of a single repeat unit in the CTD is given in the one letter code.

In this report, we present evidence that the most frequently occurring MLL fusion proteins exploit molecular control mechanisms of transcriptional elongation to transform hematopoietic cells. MLL fusions become incorporated into an “elongation assisting protein” complex, recruit it to their respective target genes, and enforce ectopic transcription. This is accompanied by DOT1L-mediated H3K79 methylation and Pol II phosphorylation through the pTEFb kinase (Figure 9). This mechanism explains and reconciles seemingly contradictory observations that have been made previously with respect to MLL fusion proteins. It has been noted that particular MLL fusion partners are almost exclusively encountered in MLL with more lymphoid characteristics, whereas others occur preferentially in the myeloid subtype. For example, MLL-AF4-transformed cells are very often of lymphatic nature. In contrast, MLL-AF9 leukemia cells are myeloid, and MLL-ENL is found in ALL, AML, and also in T-cell acute leukemia [25],[40]. These divergent phenotypes have been used as an argument against a common function for MLL fusion partners. However, the particular core structure of EAP that is stabilized by protein–protein contacts of conserved interaction domains, allows a high degree of flexibility. There are four members of the AF4 family (AF5, LAF4, and FMR2). ENL is closely related to AF9 and two CyclinT molecules (CYCT1 and CYCT2) exist in the cell. Incorporation of different homologs into the same framework might create variations of EAP that provide for cell-type or target gene specificity. Although it is generally assumed that all MLL fusions occupy identical targets, the preexisting protein environments will vary at different loci. A co-recruited EAP complex incorporating AF9 might engage in protein interactions different from those established by an EAP variant containing ENL. As a consequence, the final level of target gene activation could be dependent on the composition of the EAP. The results presented here demonstrate how the makeup of EAP is determined by the nature of the MLL fusion partner. For example, all patient-derived MLL-ENL and MLL-AF9 fusions retain the conserved C-terminus of ENL/AF9 [41],[42] that allows simultaneous recruitment of DOT1L and AF4 (or any AF4 family member) that both bridge to pTEFb. On the contrary, naturally occurring MLL-AF4/5 fusions have lost the direct pTEFb interaction domain in the N-terminus of AF4/5 [43] and need to rely on a more indirect way via ENL and DOT1L to bring in pTEFb. Structural variations in EAP and the mode of recruitment likely contribute to the observed differences in the MLL phenotypes.

As suggested by coimmunoprecipitations and RNA tethering, all protein–protein interactions that stabilize EAP seem to be conserved also in the fusion context despite the addition of an 180-kDa MLL moiety. This is corroborated also by the fact that introduction of small peptides blocking the AF4–AF9 interface has been found to be specifically toxic for MLL-AF4 cells but much less so for leukemic blasts of different etiology [44],[45]. MLL-AF4 requires AF9 (or potentially ENL) as a mediator to recruit DOT1L and pTEFb, and this pathway is blocked by binding site mimetics.

Both the positive readout in RNA tethering assays and the ChIP results indicate that MLL fusion proteins affect transcription through stimulation of elongation. In this regard, it is interesting to note that the ELL protein, the first MLL fusion partner with a known biochemical function, also is an elongation factor [46]. Later, elongation was dismissed as biochemical basis for MLL-ELL-mediated transformation because motifs in ELL important for elongation activity could be deleted in MLL-ELL with no effect for the transforming function of the protein [47]. However, it was never thoroughly tested whether domains in ELL that are essential for transformation might recruit other elongation stimulating proteins. In this regard, it will be interesting to see whether protein interaction partners of ELL [48],[49] will provide a link to elongation control. Strikingly, these ELL-associated factors (EAF1 and EAF2) have a limited but significant homology to domains in AF4 [49]. Traces of ELL have been detected in ENL precipitates [22], a possible lead that should be further explored. At present, it is hard to predict whether the more rare fusion partners will be connected to elongation control, too. This seems rather unlikely, because these proteins are mostly cytoplasmatic. However, it has been shown that the MLL fusion partner ABI1, normally also in the cytoplasm, is imported into the nucleus as MLL-ABI1 fusion due to the strong nuclear localization signals of MLL. There, ABI1 can directly interact with ENL [27], pointing to a mechanism for how cytoplasmatic fusion partners might also link to EAP and elongation control.

After initial reports to the contrary [50], it is well established that methylation of H3K79 by DOT1L is tightly associated with actively transcribed chromatin [15]. Until now, DOT1L had been implicated only in the transforming mechanism of MLL-AF10 where it could be demonstrated that interaction with DOT1L was essential for oncogenic activity of the MLL-AF10 fusion protein [17]. Here, we demonstrate a participation of DOT1L in a much wider range of MLL abnormalities encompassing the majority of all clinically observed cases. The incorporation of DOT1L in EAP also provides a molecular explanation for the genome-wide correlation of MLL-AF4 binding and a drastic increase of H3K79 methylation at the corresponding loci, a fact that has raised much interest recently [11],[12]. In addition, we show that H3K79 methylation is highly dynamic and that it is correlated with target RNA abundance. It will be interesting to know how this methyl mark is removed after MLL-ENL inactivation because no H3K79-specific demethylase has been described so far.

MLL fusion proteins are able to override normal differentiation stimuli as demonstrated by the continuing *Hoxa9* target expression and the persistent H3K79 modification of the respective locus even in cells forced to differentiate. This characterizes MLL fusions as typical “class II” oncogenic effectors that block normal maturation of precursor cells [51]. An inactivation of the fusion protein itself by pharmacological means is difficult. An inhibition of the enzymatic activities in EAP by small molecules might be a more feasible treatment option. The experiments presented here clearly show that transformation by MLL-ENL sensitizes hematopoietic cells to the effects of CDK inhibitors. This sensitivity persists in several MLL patient cell lines even after prolonged culture in vitro. In this regard, it is interesting to note that a recent report by Cleary and colleagues (Wang et al. [52]) postulated an essential role of GSK3ß for MLL fusion-mediated leukemogenesis. This is paradoxical as GSK3ß normally acts as a tumor suppressor that inactivates the Wnt pathway [53]. Therefore, GSK3ß inhibition would be expected to exacerbate the transformed phenotype. However, GSK3ß shares a 30% homology with CDK9 (unpublished data) and pharmacological GSK3ß inhibitors often also target CDKs and vice versa [54]. With regard to the involvement of CDK9 in the biochemical mechanism of MLL fusion proteins, it seems likely that at least part of the GSK3ß effect might be attributed to a concomitant block of CDK9 activity. Regardless of the contribution of each pathway, our experiments show a promising new strategy to find rational treatments for this devastating disease.

## Materials and Methods

### Plasmids, Antibodies, Cells

The cDNAs coding for either full-length or ENL mutants (also known as MLLT1, accession number NM005934), AF4 (AFF1, accession number NM_005935), AF5 (AFF4, accession number NM_014423), CYCT2 (CCNT2 accession number NM_001241), and Dot1l (accession number NM_199322) were introduced into the vectors pGADT7 or pACT2 as GAL4-activation fusions and into pGBKT7 or pAS2-1 as bait fusions (all vectors from Clontech). The human *DOT1L* cDNA is unavailable in the repositories. Therefore, the highly homolog mouse *Dot1l* was used. For RNA tethering assays, Rev fusions were constructed in pcDNA3.1-V5-HisTopoRev (gift of M. Peterlin, San Francisco, California) and tested with the pGL3-HIV1-LTR luciferase reporter plasmid. In addition, the cDNAs of ENL, AF4, AF5, CYCT2, and Dot1l were inserted into the general expression vectors pcDNA3.0 (Invitrogen) and pMSCVneo (Clontech, TaKaRa). MLL-AF5 was a kind gift of Eric So (London, United Kingdom). MLL-ENL and derivatives have been published previously [55].

Antibodies used for ChIP were from AbCam. The monoclonal anti-MLL IgM (HRX107) antibody [33] is available from Santa Cruz Biotechnology. The ENL antibody has been described [22]. All cell lines were obtained either from the DSMZ or from laboratory stocks.

### Yeast Procedures, RNA Tethering, and Luciferase Assays

For the yeast two-hybrid experiments, the bait and target plasmids were cotransfected into the yeast strain AH109 (Clontech) according to the instructions of the manufacturer (Yeast protocols handbook, Clontech). Interactions were assessed by activation of the *his* and *ade* reporter genes on synthetic media missing histidine and adenine, respectively. For a detailed description of the yeast two-hybrid procedure, see [56].

RNA tethering assays were done in 293T cells. Luciferase reporter pGL3-HIV1-LTR (0.1 µg) was cotransfected with 0.9 µg of pcDNA3-based Rev fusion plasmid by standard lipofection. Twenty-four hours after transfection, cells were lysed and luciferase was determined by standard procedures. Standard luciferase assays in Figure S2 were done with GAL4 DNA binding domain proteins on a luciferase reporter driven by a SV40 minimal promoter as described in [31]. The acidic transactivation domain (AD42) was derived from the mammalian two-hybrid vector pBAD42 (Clontech), VP16 was taken from a laboratory stock. Expression of all GAL4 constructs has been shown in [31]. Luciferase values were normalized to protein content of the cell lysates. A GFP reporter was used to control for transfection efficiencies. The normalized luciferase values were multiplied by the percentage of GFP-positive cells.

### Coimmunoprecipitations

MLL-ENL and HA-tagged versions of Dot1l or AF4 were coexpressed in 293T cells from pcDNA3-based plasmids. To detect interaction of MLL-ENL with endogenous proteins MLL-ENL was introduced alone into 293T. Analogous experiments were done for MLL-AF5 and MLL-AF4. In addition, native SEM cells containing MLL-AF4 transcribed from a natural 4;11 translocation were employed. REH cells that do not carry any 11q23 translocation were used as control. Cell extracts were prepared in 20 mM HEPES (pH 7.5), 10 mM KCl, 300 mM NaCl, 0.5 mM EDTA, 0.1% TritonX-100, and 10% glycerol supplemented with protease and phosphatase inhibitors. Anti-MLL IgM antibody (HRX107) was coupled to cyanogen bromide-activated sepharose (SIGMA), and this affinity resin was used to bring down MLL and associated proteins. Precipitates were analyzed by Western blotting for the presence of HA-tagged proteins and endogenous ENL and CDK9. For controls, the lysates were also probed with an anti-MLL antibody of a different isotype (MLL-N, mouse IgG; AbCam).

### Chromatin Immunoprecipitation, ChIP on chip

ChIP was performed according to standard procedures. In short, 10 ml (approximately 5×10e6 cells) of logarithmically growing culture was cross-linked for 10 min with 1% formaldehyde at room temperature for 10 min. Cross-linking was stopped by addition of 0.25 M glycine for a further 5 min. Cells were lysed in 50 mM Tris/HCl (pH 8.0), 10 mM EDTA, and 1% SDS, and chromatin was prepared by sonication. Precipitations were done with 2 µg of antibody and 30 µl of protein A/G agarose slurry (Santa Cruz Biotechnology) for 4 h. Precipitates were washed twice with buffer A (20 mM HEPES [pH 7.5], 10 mM KCl, 0.5 mM EDTA, 0.1% TritonX-100, and 10% glycerol) followed by two washes each in buffer A supplemented with 500 mM LiCl and buffer A + 500 mM LiCl + 0.1% SDS. After the final wash in 10 mM Tris/HCl [pH 8.0], 0.5 mM EDTA, the precipitates were eluted and cross-links were removed by incubation overnight in 50 mM Tris/HCl (pH 8.0), 10 mM EDTA, 1% SDS supplemented with 66 µg/ml RNAse A (for input samples only) and 0.5 µg of proteinase K. The treated supernatants were purified by a QIAquick Spin column (Qiagen) according to the instructions of the manufacturer. Precipitated DNA was quantified by qPCR with SYBR based premixes from Stratagene and compared to nonenriched DNA from input samples. Primer sequences used to amplify precipitated material are available on request.

For ChIP-chip experiments displayed in Figure 5, two cell lines were derived from primary mouse hematopoietic cells by transduction with flag-tagged MLL-ENL as described [57]. Chromatin IP was done as above with anti-flag agarose (M2 flag agarose, Sigma) and as a control with flag-agarose preblocked with flag peptide (Sigma). Precipitates were amplified by ligation-mediated PCR exactly as described by the manufacturer (NimbleGen-Roche) and hybridized to NimbleGen promoter MM8 RefSeqArrays. This array design tiles genomic DNA 500 bp downstream and 2 kb upstream of all known RefSeq transcripts. Data analysis was done by NimbleGen, and results were visualized by SignalMap software. The software detects potential fMLL-ENL binding sites by searching in a 500-bp sliding window for four or more peaks with a log_2_ signal-to-noise ratio above 25% of a calculated maximum that equals the average of all peaks plus six standard deviations. A false-positive discovery rate (FDR) is calculated by a 20-fold randomization of the ratio data, and a probability of “randomness” is assigned to each peak. Peaks with a probability of <0.2 are indicative for binding. In total, two independent experiments were performed.

### Inhibitor Tests with Primary Cells

Six- to 8-wk-old Balb/C mice were treated by intraperitoneal administration of 150 mg/kg 5-fluorouracil. Five days after injection, bone marrow enriched in precursor cells was harvested. Half of the cells were cultivated for 3 to 4 d in RPMI medium supplemented with 10 ng/ml IL-3, IL-6, and GM-CSF, as well as with 100 ng/ml SCF (all recombinant cytokines were obtained from PeproTech) and with increasing amounts of alsterpaullone or flavopiridol (Sigma). Proliferation was assessed by a standard MTT assay. The second batch of cells was retrovirally infected with MLL-ENL as described in [57], and after selection for transduced cells, the resulting MLL-ENL-transformed lines were checked for sensitivity towards alsterpaullone and flavopiridol under identical cytokine conditions as used for nontransformed cells.

## Supporting Information

Figure S1 **Two-hybrid experiments.** (A) Two-hybrid pairings. A series of deletion mutants derived from ENL, AF4, CYCT2A, and Dot1l was tested in two-hybrid assays with full-length proteins as interaction partner. Numbers correspond to amino acid residues. Abbreviations are as in Figure 1. Two-hybrid outcome is listed either as + =  strong interaction, (+) =  weak interaction, growth only after prolonged incubation, or − =  no interaction. (B) Expression of two-hybrid clones. Extracts of transformed yeast cells were blotted and probed with a GAL4-DNA binding domain-specific antibody. Expression from plasmids not listed here has been shown previously [20],[26],[27].
(3.48 MB TIF)

Figure S2 **Transactivation potential and elongation stimulation by ENL, AD42, and VP16.** ENL as well as the generic transactivation domains AD42 (acidic transactivation domain derived from a mammalian two-hybrid vector) and VP16 (a transactivator domain from *H. simplex*) were fused to the GAL4 DNA-binding domain and to Rev. (A) General transactivation potential of GAL4 recruited proteins. GAL4 fusions were tested on a SV40 minimal promoter-based luciferase reporter as described in [31]. Depicted are average values and standard deviations of triplicate transfections. The expression of the corresponding GAL4-proteins has been shown in Zeisig et al. [31]. The green bar highlights results obtained for the fusion of GAL4 with ENL. (B) Rev fusions of the same proteins were examined for their elongation stimulation activity in the TAR-loop RNA tethering assay. The expression of the respective Rev fusions is demonstrated by a Rev-specific Western blot. Values are charted as described for (A).
(1.42 MB TIF)

Figure S3 **Kinetics of RNA Pol II activity on** ***Hoxa9*** **chromatin.** (A) Schematic depiction of the putative *Hoxa9* core transcript as annotated in Ensembl (ENSMUST00000048680). Primers used for ChIP and for qRT-PCR are indicated. (B) ChIP and RNA decay kinetics. ChIP was performed on cells transformed by inducible MLL-ENL as described for Figure 6. Samples were taken in the presence of tamoxifen (active MLL-ENL) and at the indicated time points after withdrawal of the inductor. ChIP was performed with antibodies specific for the serine-2 and the serine-5 phosphorylated isoforms of RNA Pol II and RNA was extracted, digested with DNAseI, and reverse transcribed into cDNA. ChIP precipitates were quantified in relation to input samples by qPCR with the primers indicated in (A). Data are plotted as relative values compared to day 0. cDNA was analyzed by qPCR, and data were normalized to ß-actin. In addition to the 5′ and 3′ primers that would detect unspliced and spliced RNA, the intron-spanning primer is specific for spliced *Hoxa9* transcripts.
(0.87 MB TIF)

## Abbreviations

ChIP: chromatin immunoprecipitation
MLL: mixed-lineage leukemia
qRT-PCR: quantitative reverse-transcriptase PCR
