# Supplementary figures and images for: Misguided Transcriptional Elongation Causes Mixed Lineage Leukemia
Source: PLoS Biol. 2009 Nov 24;7(11):e1000249. doi: 10.1371/journal.pbio.1000249 (PMC2774266; doi:10.1371/journal.pbio.1000249)

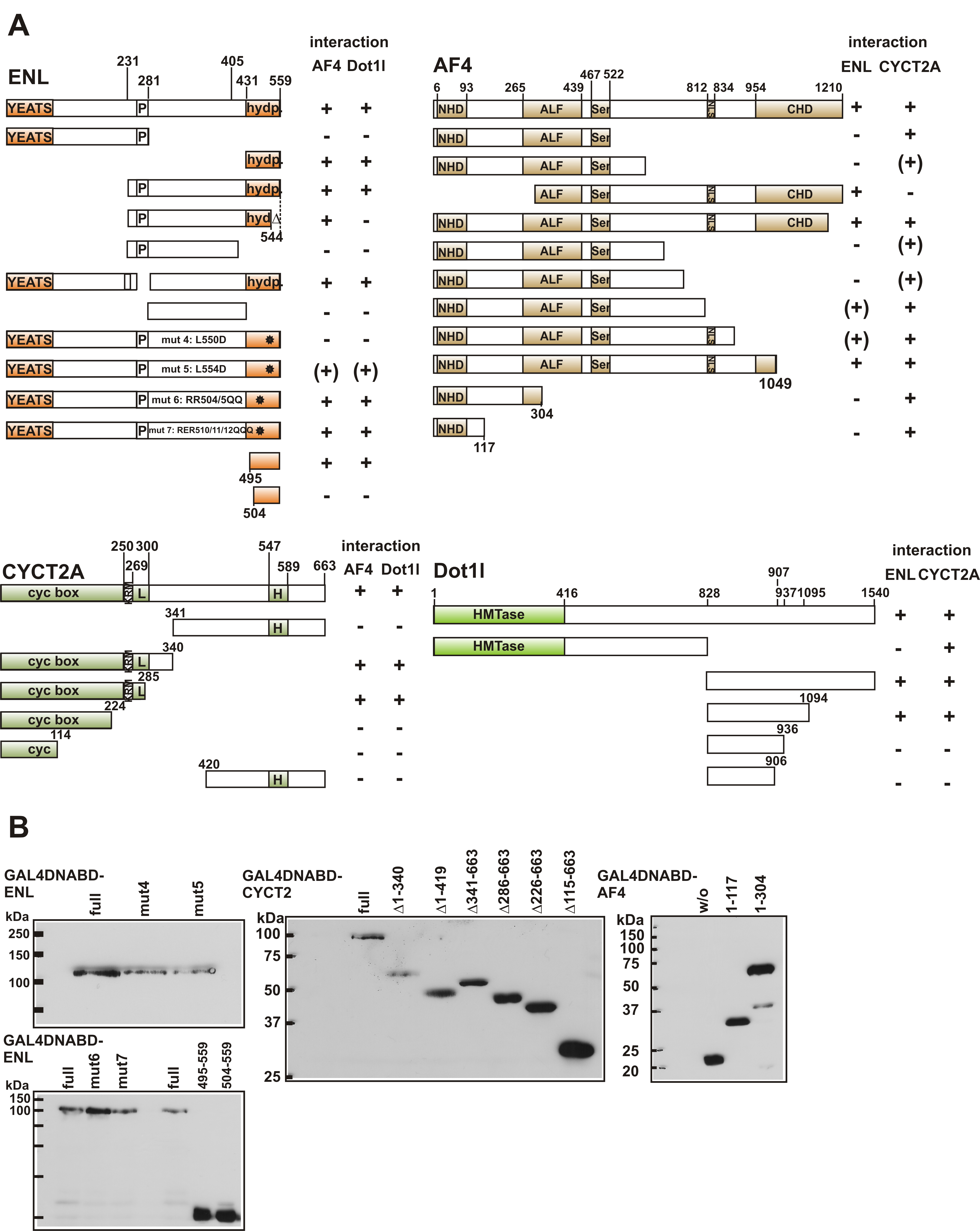

Supplement: Figure S1 — Two-hybrid experiments. (A) Two-hybrid pairings. A series of deletion mutants derived from ENL, AF4, CYCT2A, and Dot1l was tested in two-hybrid assays with full-length proteins as interaction partner. Numbers correspond to amino acid residues. Abbreviations are as in Figure 1. Two-hybrid outcome is listed either as + = strong interaction, (+) = weak interaction, growth only after prolonged incubation, or − = no interaction. (B) Expression of two-hybrid clones. Extracts of transformed yeast cells were blotted and probed with a GAL4-DNA binding domain-specific antibody. Expression from plasmids not listed here has been shown previously [20],[26],[27]. (3.48 MB TIF) [file pbio.1000249.s001.tif]

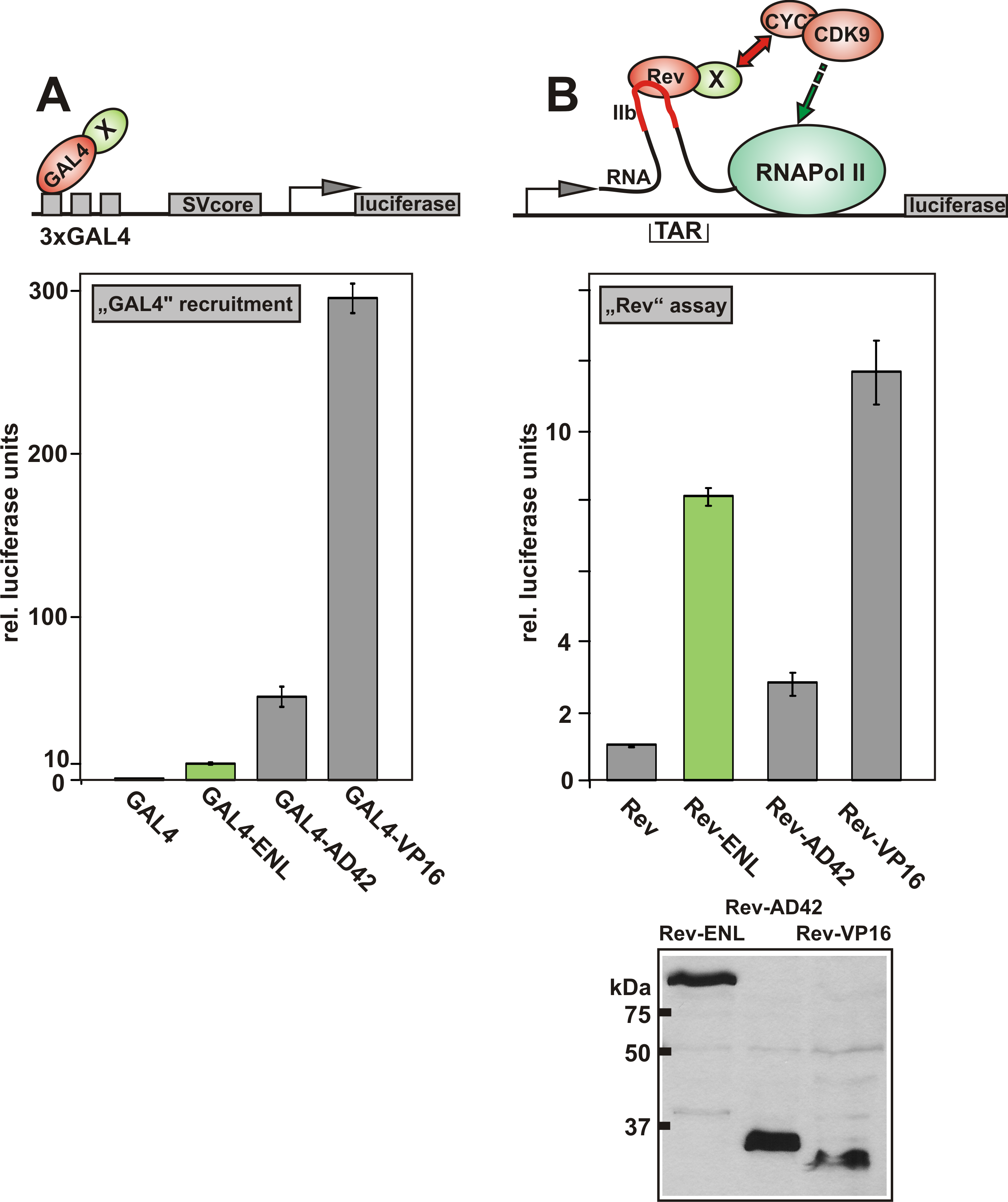

Supplement: Figure S2 — Transactivation potential and elongation stimulation by ENL, AD42, and VP16. ENL as well as the generic transactivation domains AD42 (acidic transactivation domain derived from a mammalian two-hybrid vector) and VP16 (a transactivator domain from H. simplex) were fused to the GAL4 DNA-binding domain and to Rev. (A) General transactivation potential of GAL4 recruited proteins. GAL4 fusions were tested on a SV40 minimal promoter-based luciferase reporter as described in [31]. Depicted are average values and standard deviations of triplicate transfections. The expression of the corresponding GAL4-proteins has been shown in Zeisig et al. [31]. The green bar highlights results obtained for the fusion of GAL4 with ENL. (B) Rev fusions of the same proteins were examined for their elongation stimulation activity in the TAR-loop RNA tethering assay. The expression of the respective Rev fusions is demonstrated by a Rev-specific Western blot. Values are charted as described for (A). (1.42 MB TIF) [file pbio.1000249.s002.tif]

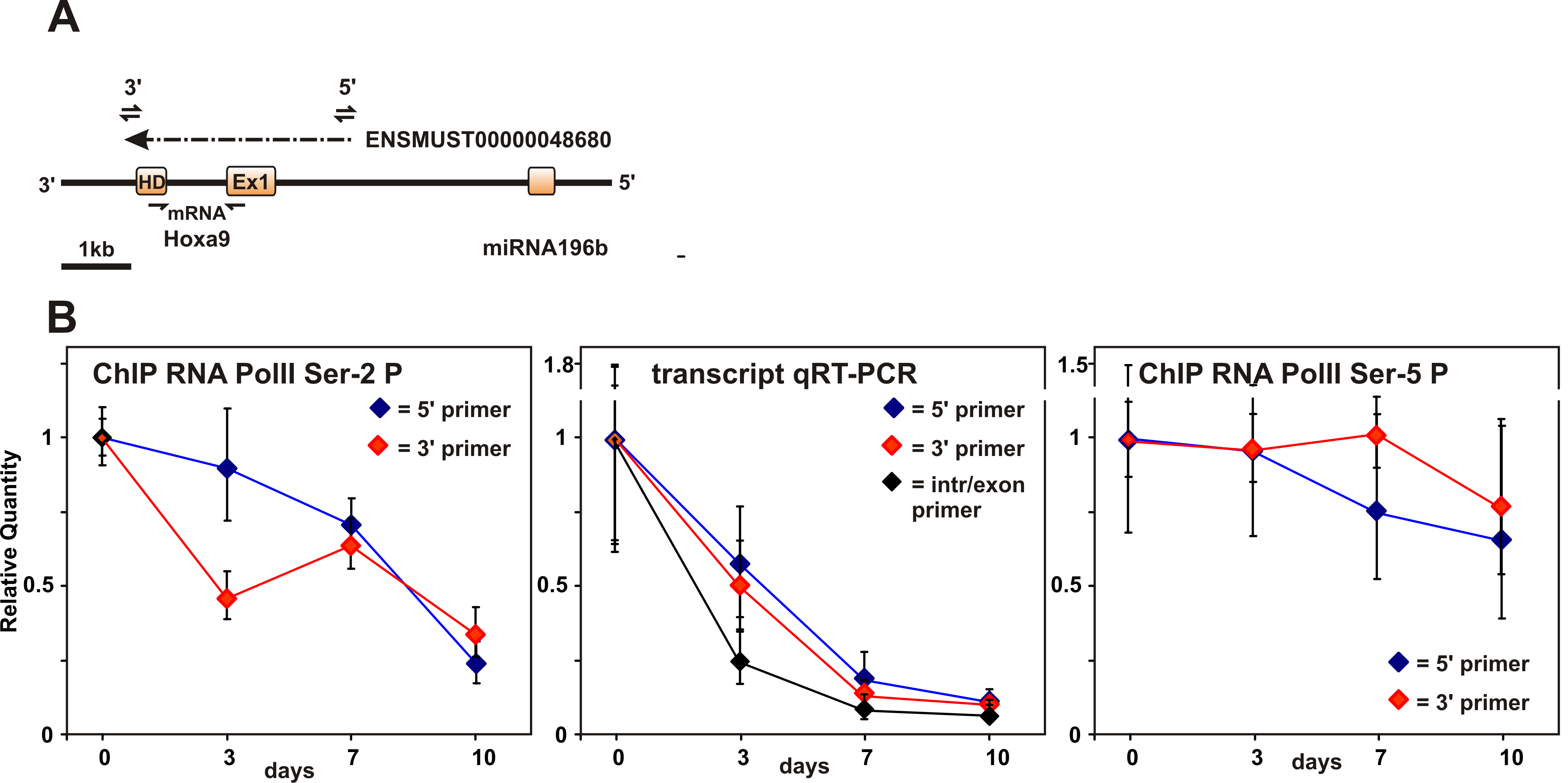

Supplement: Figure S3 — Kinetics of RNA Pol II activity on Hoxa9 chromatin. (A) Schematic depiction of the putative Hoxa9 core transcript as annotated in Ensembl (ENSMUST00000048680). Primers used for ChIP and for qRT-PCR are indicated. (B) ChIP and RNA decay kinetics. ChIP was performed on cells transformed by inducible MLL-ENL as described for Figure 6. Samples were taken in the presence of tamoxifen (active MLL-ENL) and at the indicated time points after withdrawal of the inductor. ChIP was performed with antibodies specific for the serine-2 and the serine-5 phosphorylated isoforms of RNA Pol II and RNA was extracted, digested with DNAseI, and reverse transcribed into cDNA. ChIP precipitates were quantified in relation to input samples by qPCR with the primers indicated in (A). Data are plotted as relative values compared to day 0. cDNA was analyzed by qPCR, and data were normalized to ß-actin. In addition to the 5′ and 3′ primers that would detect unspliced and spliced RNA, the intron-spanning primer is specific for spliced Hoxa9 transcripts. (0.87 MB TIF) [file pbio.1000249.s003.tif]
